# Artificial neural networks allow the use of simultaneous measurements of Alzheimer Disease markers for early detection of the disease

**DOI:** 10.1186/1479-5876-3-30

**Published:** 2005-07-27

**Authors:** Monica Di Luca, Enzo Grossi, Barbara Borroni, Martina Zimmermann, Elena Marcello, Francesca Colciaghi, Fabrizio Gardoni, Marco Intraligi, Alessandro Padovani, Massimo Buscema

**Affiliations:** 1 Centre of Excellence for Neurodegenerative Disorders and Department of Pharmacological Sciences, University of Milan, Italy; 2 Medical Department, Bracco Spa, Milan, Italy; 3 Department of Neurological Sciences, University of Brescia, Italy; 4 Centro Ricerche Semeion, Rome, Italy

## Abstract

**Background:**

Previous studies have shown that in platelets of mild Alzheimer Disease (AD) patients there are alterations of specific APP forms, paralleled by alteration in expression level of both ADAM 10 and BACE when compared to control subjects. Due to the poor linear relation among each key-element of beta-amyloid cascade and the target diagnosis, the use of systems able to afford non linear tasks, like artificial neural networks (ANNs), should allow a better discriminating capacity in comparison with classical statistics.

**Objective:**

To evaluate the accuracy of ANNs in AD diagnosis.

**Methods:**

37 mild-AD patients and 25 control subjects were enrolled, and APP, ADM10 and BACE measures were performed. Fifteen different models of feed-forward and complex-recurrent ANNs (provided by Semeion Research Centre), based on different learning laws (back propagation, sine-net, bi-modal) were compared with the linear discriminant analysis (LDA).

**Results:**

The best ANN model correctly identified mild AD patients in the 94% of cases and the control subjects in the 92%. The corresponding diagnostic performance obtained with LDA was 90% and 73%.

**Conclusion:**

This preliminary study suggests that the processing of biochemical tests related to beta-amyloid cascade with ANNs allows a very good discrimination of AD in early stages, higher than that obtainable with classical statistics methods.

## Introduction

Neurodegenerative diseases as Alzheimer Disease (AD) are posing a tremendous impact on our society. There is no remission in the progression, and pharmacological interventions available at present require an early and accurate identification of the disease [1].

Up to now, the diagnosis of probable and possible AD is based on neuropsychological evaluation by multidimensional assessments. Differential diagnosis between AD and other types of dementia is often based on exclusion [2].

Thus, the possibility to use a biomarker supporting clinical diagnosis would be of great relevance in the early detection of the disease.

For early AD diagnosis, an ideal diagnostic tool must be sensitive to earlier cognitive and biological changes, but is should be able to differentiate among AD, normal aging and other forms of dementia or pseudo dementia. It should be reliable, readily applicable and simple [3]. In the last years, we have identified in easily accessible circulating cells, i.e. platelets, a combination of biological measurements that possess all characteristic to be considered as highly accurate biomarkers for AD [4-6]. In platelets it is possible to measure the levels of three molecular identities key-elements in the amyloid-cascade, namely Amyloid Precursor Protein (APP) forms as well as beta-secretase (beta-site- APP cleaving enzyme, BACE1) enzyme, responsible for amyloidogenic pathway, and alpha-secretase (ADAM10) responsible for non-amyloidogenic metabolism [7,8]. Further, we have demonstrated a concomitant and congruent modification of these biochemical parameters in platelets of AD patients when compared to control subjects [6]. Indeed, in platelets of mild AD patients we were able to show an alteration of specific APP forms, paralleled by a decreased expression level and activity of ADAM 10 as well as an increased BACE activity, when compared to control subjects [8].

The simultaneous measurement of these biochemical parameters can be considered as a useful "combining strategy" to enhance the accuracy of the biological testing. However, this approach has intrinsic constrains, related to the statistical analysis used since classical statistics approaches suffer from underlying non linearity among variables.

Artificial Neural Networks (ANNs) are adaptive models for the analysis of data which are inspired by the functioning processes of the human brain. They are systems which are able to modify their internal structure in relation to a function objective. They are particularly suited for solving problems of the non linear type, being able to reconstruct the approximate rules that put a certain set of data – which describes the problem being considered – with a set of data which provides the solution [9].

Thus, the aim of this study was to assess the efficacy of neural network in correctly classifying control subjects and mild AD patients only on the basis of peripheral beta-amyloid cascade biomarkers.

## Methods

### Subjects

The study was carried out on 37 probable mild AD patients, and 25 healthy age-matched controls (CON), in accordance with local clinical research regulations and an informed consent was obtained. Each subject underwent a clinical and a standardized neuropsychological assessment for evaluation of cognitive functions, activities of daily living and behavioral and psychological disturbances.

Probable AD diagnosis was based on National Institute of Neurological and Communicative Disorders and Stroke-Alzheimer's Disease and Related Disorders Association criteria (NINCDS-ADRDA) [10]. Severity of dementia was rated according to Clinical Dementia Rating (CDR) scale, and only patients with CDR≤1 entered the study.

The following exclusion criteria were applied: a) major depressive disorder, bipolar disorder, schizophrenia, substance use disorder, or mental retardation according to DSM-IV criteria; b) cerebro-vascular disorder, hydrocephalus, and intra-cranial mass, documented by CT or MRI within the last 12 months; c) abnormalities in serum folate and vitamin B12, syphilis serology, or thyroid hormones' levels; d) a history of traumatic brain injury or other neurological disease; e) significant medical problems (e.g. diabetes or hypertension; cancer; hepatic, renal, cardiac or pulmonary disorders).

Further, subjects on psychotropic agents, nootropic drugs, cholinergic or anticholinergic agents, antiplatelets agents, anticoagulants, steroids, and serotoninergic drugs, were excluded unless they entered a wash-out phase lasting at least 14 days before blood collection.

### Platelets collection and Western blot analysis

Blood drawing occurred while fasting at 9.00–10.00 AM. A blood sample (27 ml) was taken, releasing the tourniquet, using a 19-gauge needle, and processed as previously described (6). Platelets were processed for Western blot analysis (WB) with monoclonal antibody (mAb) 22C11 dilution 1:2000 (Chemicon, Tamecula) raised against the N-terminal domain of APP, therefore recognizing the three APP forms with apparent molecular weight of 130, 110, and 106 kDa. The results were expressed as the ratio (APP ratio) of the optical density of the upper (130 kDa) to the lower (106–110 kDa) immunoreactive bands. WB was also performed with polyclonal antibody (pAb) to ADAM10 dilution 1:1000 (Proscience Inc., Poway, CA USA); pAb to BACE 1:500 (Affinity Bioreagents Inc., Golden, CO USA), clone AC40 for β-ACTIN 1:3000 (Sigma-Aldrich Steinheim, Germany) were used. Immunostaining β-ACTIN, a constitutive protein, was used as internal standard. The OD between ADAM 10 and β-ACTIN was measured. Similarly, BACE analysis was performed, and the OD of the two forms (BACE 36 kDa/BACE 57 kDa) was evaluated.

Quantitative analysis of WB was performed by means of computer assisted imaging (Quantity-OneR System; Biorad, CA, USA).

### Artificial Neural Networks

In this study, we applied supervised ANNs, networks in which the result of the processing (the output desired) is already defined. Supervised ANNs calculate an error function that measures the distance between the desired fixed output (target) and their own output, and adjust the connection strengths during the training process to minimize the result of the error function. The learning constraint of the supervised ANNs is having their own output coincide with the determined target. The general form of these ANNs is: y = f(x,w*), where w* constitutes the set of parameters which best approximate the function. The ANNs used in the study are characterized by the law of learning and topology. The laws of learning identify equations which translate the ANNs inputs into outputs, and rules by which the weights are modified to minimize the error or the internal energy of the ANNs.

The topology identifies the structure of the nodes of ANNs connections and the signal's flow within it. ANNs can be further distinguished by two broad categories: feed-forward ANNs (FF), in which the signal proceeds from the input to the output of the ANNs, crossing all of the nodes only once; recurring ANNs, in which the signal is subject to specific feedback, determined beforehand, or tied to the occurrence of particular conditions.

The experiments carried out anticipate the use of both ANNs and Artificial Organisms, i.e. complex combinations of more networks. Supervised software (Semeion ^©^), which allows the combination of each law of learning with each type of topology, was used for all the testing.

Four models of ANNs and one statistical model used in the study, as follows.

1) The *Back Propagation *standard (BP-FF) is defined by different interconnected layers of nodes characterized by a non linear function, generally of the sigmoidal type [11].

2) The *Sine Net *(SN) (Semeion ^©^) is characterized by the organization of nodes in layers [12]. The fundamental difference with respect to the better known BP-FF is due to the characteristics of the function realized by each single node. In fact, the node activation function, which has similar characteristics to those of the BP-FF, operates on an input that is made up of a sum of sines, each characterized by its own frequency (the weight of the link/connection). This modification on the base structure of the node has profound consequences on the behaviors of the SN both for the function's calculated properties and for the learning process modalities.

3) The *Bi-Modal *networks (BM) (Semeion ^©^) are multi-layered networks like Sn and Bp, but differ from these because of the equations which characterize them [13]. Each hidden node is realized by 2 sub-nodes, each equipped with its own input connections: the first sub-node operates according to the descending gradient technique, the second through vectorial quantification. The outputs of the two nodes are then composed in a single output value. These networks therefore originate from the hybridization of the two most relevant learning mechanisms in the sphere of artificial intelligence: the descending gradient and the vectorial quantification. The Bi-Modal demonstrates an excellent convergence capacity on complex problems; furthermore it does not suffer from problems on local minimums shown by other algorithms, which use the principal of the descending, gradient exclusively.

4) The *Soft Max Discriminant Analysis *(SMDA) is an ANNs model composed by a single layer of units, without the layer of hidden units, where the Soft-Max function is applied to the output layer.

### Statistical models

Results obtained with the neural networks cited above have been compared with a model of linear statistic, i.e. the Linear Discriminant Analysis (LDA) (Software SPSS^®^).

The validation protocol is a fundamental procedure to verify the models' ability to generalize the results reached in the testing phase. Among the different protocols reported in literature, the selected model is the protocol with the greatest generalization ability on data unknown to the model itself. The dataset was randomly subdivided into two sub-samples: the first called Training Set, and the second, called Testing Set; a fixed ANN (and/or Organism) which is trained on the Training Set was chosen. In this phase, the ANN learns to associate the input variables with those that are indicated as targets; the weight matrix produced by the ANNs at the end of the training phase was saved, and freezed with all parameters used for the training; finally, each case belonging to the Testing Set was shown to the ANN, in order to allow the ANN to express an evaluation based on the training just performed. This procedure takes place for each input vector but every result (output vector) is not communicated to the ANN; in this way, the ANN is evaluated only in reference to the generalization ability that it has acquired during the Training phase.

Then, a new ANN with identical architecture to the previous one was constructed and the procedure from point 1 was repeated.

This general training plan has been further developed to increase the level of reliability of the generalization of the processing models. The experiments have been done using a random criterion of distribution of the samples. We have employed the 5 × 2 cross-validation protocol, which produces 10 elaborations for every sample. It consists in dividing the sample five times in two sub samples, containing each similar distribution of cases and controls [14].

## Results

### Descriptive Analysis

Control subjects and mild AD patients were comparable as far as age and gender distribution (see Table 1).

**Table 1:** Demographic, clinical and biological variables of the subjects.

***Variables***	**CONTROLS**	**Mild AD**	***p***
Number	25	37	-
Age, years	66.5 ± 3.8	67.3 ± 6.8	*n.s.*
Gender, M/F	11/14	17/20	*n.s.*
MMSE score	29.6 ± 1.0	24.9 ± 4.4	*.0001*
CDR score	-	0.7 ± 0.28	*.0001*
APPr	0.61 ± 0.22	0.44 ± 0.18	*.003*
BACE	1.52 ± 1.27	0.84 ± 0.73	*.008*
ADAM 10	0.79 ± 0.49	0.43 ± 0.23	*.0004*

M: male; F: female; MMSE: Mini-Mental State Examination; CDR: Clinical Dementia Rating; APPr: Amyloid Precursor Protein ratio.

**Figure 1:**
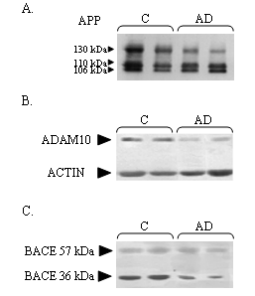
Representative Western Blot analysis of APP forms (panel A), ADAM10 (panel B), and BACE (panel C) in AD patients and in healthy controls.

Figure 1 reports a representative Western Blot of APP forms, ADAM10, and BACE levels in 2 control subjects and in 2 mild AD patients, the latter showing an alteration of these three measures. As shown, the optical density of the upper (130 kDa) APP form was reduced in AD patients compared to controls, being APP ratio decreased in the former group (**panel A**). Moreover, a reduction in the optical density ratio between ADAM10 and internal standard control (actin) as well as between the 36 kDa and the 57 kDa BACE forms was demonstrated in AD (see **panel B,C**). A significant statistical difference was present in APP ratio, BACE and ADM10 values between the two groups (see Table 1).

The statistical significance of the differences reached an higher value for ADAM 10 in comparison with BACE and APP respectively, essentially due to a minor coefficient of variation index and a higher correlation index value (R^2 ^= 0.012) with the target variable (diagnostic class).

Despite these average differences there was a certain degree of overlap of beta amyloid markers values across the two study groups, which made difficult the precise distinction among individual subjects belonging to one of the two classes.

### ANNs Analysis

In this study two kinds of protocols with neural networks models have been carried out in order to distinguish individual control subjects from mild AD patients only on the basis of APP, BACE1 and ADAM 10 values. In the first protocol, which included 170 analyses, APP, BACE and ADAM10 constituted the input variables. In the second protocol, which included 150 analyses, the three possible dual combinations of parameters constituted the input variables (APP – BACE; APP – ADAM10; BACE – ADAM)

Table 2 summarizes the average results related to 10 independent analyses with each different ANN model using three input variables and 5 × 2 cross-validation protocol.

The best result was obtained with Self Recurrent Network Dynamic Sine Net (SelfDASn) model, which reached an overall arithmetic mean accuracy rate of 93.08%. The corresponding result obtained with Linear Discriminant Analysis (LDA) was equal to 81.6%.

In Table 3 the average results related to 10 independent analyses with each different ANN model using two input variables and 5 × 2 cross-validation protocol are reported. In these analyses, Feed-Forward ANN models based on three learning laws (Back-Propagation, SineNet e BiModal) have been employed in order to define more precisely the contribution of each marker in discriminating AD.

**Table 2:** Three input variables analyses and 5 × 2 cross-validation protocol (average results obtained in 10 analyses with each model). LDA: Linear Discriminant Analysis, No: Number; SD: Standard Deviation.

**MODELS**	**No.**	**Senitivity**	**Specificity**	**Mean Accuracy**	**No. Errors**	**SD Mean Accuracy**	**p**
***SelfDASn***	10	94,03%	92,12%	**93,08%**	2,1	2,98	0,000036
***TasmDASn***	10	95,12%	90,51%	92,82%	2,1	3,26	0,000016
***TasmSASn***	10	92,51%	91,99%	92,25%	2,4	2,67	0,000022
***SelfSASn***	10	94,50%	89,87%	92,19%	2,3	2,58	0,000020
***TasmDABm***	10	93,54%	88,91%	91,23%	2,6	2,84	0,000007
***TasmDABp***	10	93,51%	88,91%	91,21%	2,6	3,20	0,000001
***SelfSABm***	10	93,57%	88,14%	90,86%	2,7	3,76	0,000144
***SelfDABp***	10	92,43%	88,14%	90,29%	2,9	3,35	0,000031
***TasmSABp***	10	90,79%	88,98%	89,88%	3,1	3,14	0,000302
***SelfDABm***	10	91,34%	88,21%	89,78%	3,1	4,02	0,000398
***TasmSABm***	10	91,90%	87,37%	89,64%	3,1	4,07	0,000504
***SelfSABp***	10	90,29%	88,91%	89,60%	3,2	2,96	0,000311
***FF_Sn***	10	91,90%	84,17%	88,03%	3,5	4,11	0,000734
***FF_Bm***	10	88,63%	86,54%	87,58%	3,8	3,61	0,000860
***FF_Bp***	10	88,63%	85,77%	87,20%	3,9	3,72	0,001092
***SMDA***	10	89,71%	78,65%	84,18%	4,6	6,31	0,021734
**LDA**	10	90,26%	72,95%	**81,61%**	5,2	5,47	-

**Table 3:** Two variables analyses and 5 × 2 cross-validation protocol (average results obtained in 10 analyses with each model).

**INPUT**	**MODELS**	**No.**	**Sensitivity**	**Specificity**	**Mean Accuracy**	**No. Errors**	**SD Mean Accuracy**
**APP ADAM**	***FF_Sn***	10	92,98%	76,99%	84,99%	4,2	4,23
	***FF_Bm***	10	93,51%	73,08%	83,29%	4,6	6,06
	***FF_Bp***	10	89,12%	74,74%	81,93%	5,2	5,27
	***SMDA***	10	87,98%	69,17%	78,58%	6,1	6,12
	***LDA***	10	82,52%	63,46%	72,99%	7,8	8,74

**APP BACE**	***FF_Sn***	10	84,33%	80,06%	82,20%	5,4	3,53
	***FF_Bm***	10	81,69%	79,94%	80,82%	5,9	4,24
	***FF_Bp***	10	83,27%	79,17%	81,22%	5,7	3,92
	***SMDA***	10	82,81%	76,60%	79,70%	6,1	4,21
	***LDA***	10	79,53%	64,68%	72,11%	8,2	4,54

**ADAM BACE**	***FF_Sn***	10	86,52%	80,19%	83,36%	5	5,91
	***FF_Bm***	10	86,02%	76,92%	81,47%	5,5	5,60
	***FF_Bp***	10	84,88%	79,42%	82,15%	5,4	6,61
	***SMDA***	10	83,86%	74,61%	79,24%	6,2	7,05
	***LDA***	10	85,97%	63,53%	74,75%	7,2	6,76

LDA: Linear Discriminant Analysis, No: Number; SD: Standard Deviation.

The comparison with LDA was performed as well, being the accuracy of ANN higher. The differential performance rate between ANNs and LDA was the following 12% for APP+ADAM (FF-Sn 84.99% – LDA 72.99%), 10.09% for APP+BACE (FF-Sn 82.20% – LDA 72.11%), and 8.61% for ADAM+BACE (FF-Sn 83.36% – LDA 74.75%).

In the three variables protocol, the corresponding differential value obtained with the best feed forward ANNs was equal to 6.42% (FF-Sn 88.03% – LDA 81.61%)

### Input relevance analysis

In addition to evaluate the overall performance rate in discriminating between target classes, the AAN models allow the definition of the net relevance of each input in the occurring model. This evaluation has been performed using the seven best performers ANN models (overall accuracy higher than 97% in blind testing). As reported in Table 4, the input relevance value referred to each independent marker was reported. APP ratio resulted to be the most relevant variable in one model, thus reaching the 41%, while ADAM10 and BACE showed the highest input relevance three times each.

**Table 4:** The input relevance value referred to each independent marker (in bold).

					***Input***	***relevance***	***values***
					
**ANN model**	**Sensitivity**	**Specificity**	**Mean Accuracy**	**No. errors**	**APP**	**BACE**	**ADAM**
***SelfDASn***	94,74%	100,00%	97,37%	1/32	**0,4155**	0,3001	0,2845
***TasmDASn***	94,74%	100,00%	97,37%	1/32	0,2528	0,2445	**0,5026**
***SelfDABm***	94,44%	100,00%	97,22%	1/30	0,2802	**0,4074**	0,3124
***SelfDABp***	94,44%	100,00%	97,22%	1/30	0,2084	**0,4285**	0,3631
***SelfDASn***	94,44%	100,00%	97,22%	1/30	0,3653	0,2598	**0,3749**
***SelfSABm***	94,44%	100,00%	97,22%	1/30	0,2769	**0,4273**	0,2958
***SelfSASn***	94,44%	100,00%	97,22%	1/30	0,3577	0,2486	**0,3937**

## Discussion

The main finding of this study is that the application of artificial neural networks on a set of biomarkers for AD, designed on the amyloid cascade, improved by more than 10% the accuracy of the early diagnosis of the disease.

The potential medical application of diagnostic biomarkers has generated much excitement within the biomedical community for early disease diagnosis in AD. Several biomarkers have been proposed in the last years as promising candidates to assess AD susceptibility among individual at risk for the disease.

In recent years, we have assisted to the transition from a single biomarker to a multiple biomarkers approach [15]. The simultaneous use of different biomarkers, each of them exploring a component of a complex patho-physiological pathway, has the intrinsic advantage to capture more information linked to the disease under study compared to a single biomarker approach. The concomitant use of different factors may decrease the risk of random variation of single factor obscuring the true signal.

Many medical decisions are made in situations in which multiple factors must be weighted. Because multiple predictive features may interact and correlate with outcomes in complex ways, effective statistical and modeling tools are needed to integrate these data and to determine their implications. This is also true for biomarkers use. When single factor approach is applied to the analysis of multi-markers data, only one factor (marker) is varied at time, with the others factors held constant. This is the case of classical multivariable statistical techniques. With these techniques the combined interpretation of a given set of potential predictors with respect to individual patients may be difficult. This is mainly due to the limitations imposed by the underlying non-linearities and to complex interactions between the factors under study.

Neural networks can input multiple factors simultaneously, combining and recombining them in different ways according to specific (generally non linear) equations. Thus, the higher predictive values obtained by AAN could be explained because of the consideration of parameters which might not reach significance for the entire population, but are highly significant within subgroups. On the other hand, the conventional statistics reveal only parameters which are significant for the entire population.

The adaptive systems had superior accuracy when compared to models of classic linear statistics. In fact, assessing the presence of AD, ANNs were more efficient than conventional statistical analysis, e.g. discriminant analysis, achieving a correct performance (diagnosis) in extremely high percentage of subjects on average and reaching a predictive accuracy of 93% when the best net was used.

Recently, the hypothesis to use a combination of biochemical tests in order to improve the discrimination of AD in early stages has been put forward [8].

The use of ANNs for detecting association between biological markers and disease susceptibility has recently attracted the attention of the scientific and clinical community [16,17]. In fact, to enhance diagnostic accuracy combining tests should be considered to increase the discriminative power of the analysis. In this regard, two different strategies might be followed: either combining tests related to different patho-physiological pathways or associating biomarkers linked to the same biological cascade.

Here we showed that AAN models allow to evaluate three key-elements of amyloid cascade for diagnostic purpose, reaching sensitivity and specificity values higher than those obtained in clinical practice. Studies performed on a large sample with very mild AD patients, pre-symptomatic subjects or patients with other kind of dementia, will be useful to confirm the diagnostic value of this approach.
